# Description of *Hymenolepis microstoma *(Nottingham strain): a classical tapeworm model for research in the genomic era

**DOI:** 10.1186/1756-3305-3-123

**Published:** 2010-12-31

**Authors:** Lucas J Cunningham, Peter D Olson

**Affiliations:** 1 Department of Zoology, The Natural History Museum, Cromwell Road, London, SW7 5BD, UK

## Abstract

**Background:**

*Hymenolepis microstoma *(Dujardin, 1845) Blanchard, 1891, the mouse bile duct tapeworm, is a rodent/beetle-hosted laboratory model that has been used in research and teaching since its domestication in the 1950s. Recent characterization of its genome has prompted us to describe the specific strain that underpins these data, anchoring its identity and bringing the 150+ year-old original description up-to-date.

**Results:**

Morphometric and ultrastructural analyses were carried out on laboratory-reared specimens of the 'Nottingham' strain of *Hymenolepis microstoma *used for genome characterization. A contemporary description of the species is provided including detailed illustration of adult anatomy and elucidation of its taxonomy and the history of the specific laboratory isolate.

**Conclusions:**

Our work acts to anchor the specific strain from which the *H. microstoma *genome has been characterized and provides an anatomical reference for researchers needing to employ a model tapeworm system that enables easy access to all stages of the life cycle. We review its classification, life history and development, and briefly discuss the genome and other model systems being employed at the beginning of a genomic era in cestodology.

## Background

Species of *Hymenolepis *Weinland, 1858 (Platyhelminthes: Cestoda: Cyclophyllidea) have been used as tapeworm models in research and teaching since the 1950s when they were first domesticated in the laboratory of Clark P. Read [1]. Adult parasites of rodents with beetle intermediate hosts, they benefit from easy culture *in vivo *using natural hosts that are themselves model organisms (e.g. *Mus musculus* L., *Tribolium confusum *Jacquelin du Val). Research on *Hymenolepis*, and especially *H. diminuta *(Rudolphi, 1819), *H. nana *(von Siebold, 1852) and *H. microstoma*, is underpinned by an extensive literature that includes much of our classical knowledge of tapeworm biology [e.g. [2]]. A recently initiated effort sponsored by The Wellcome Trust Sanger Institute to characterize the genome and adult and larval transcriptomes of *H. microstoma *http://www.sanger.ac.uk/sequencing/Hymenolepis/microstoma/ has brought this classical model into the genomic era, greatly advancing its utility for researchers interested in employing a practical tapeworm system that allows access to all life cycle stages. In light of this development, and the fact that laboratory isolates can vary in features of their biology [3], it is desirable to have a description of the exact strain on which the genome is based, and to thus anchor the data to a well-defined entity.

*Hymenolepis microstoma *was first described from the bile ducts of mice in 1845 by Dujardin [4] who placed it in the genus *Taenia *L., 1758, which housed all tapeworms known at that time. In 1891, Blanchard [5] transferred the species to the genus *Hymenolepis *and provided an expanded description of the species. Although Bear and Tenora [6] suggested synonymy between *H. microstoma *and *H. straminea *(Goeze, 1782), species status of *H. microstoma *historically has been widely accepted, and molecular data have shown both species to represent independent, albeit closely related, lineages [7,8]. In contrast, the genus *Hymenolepis *has itself been overhauled on several occasions and its membership and internal structure remain controversial. For example, whereas Hughes [9,10] accepted the generic assignment *H. microstoma *by Blanchard, Spasskii [11] subdivided the genus and transferred *H. microstoma *to the genus *Rodentolepis *Spasskii, 1954, which he erected to house the rodent-hosted species of *Hymenolepis *with armed rostella. At the same time Spasskii erected the genus *Vampirolepis *Spasskii, 1954, which Schmidt subsequently considered a senior synonym of *Rodentolepis*, thus resulting in the new combination *Vampirolepis microstoma *(Dujardin, 1854) Schmidt, 1986 [12]. The genus *Rodentolepis *was retained by Czaplinski and Vaucher [13] in the most recent synoptic treatment of tapeworms [14], but this work did not consider species level taxa and therefore did not arbitrate on the generic assignment of *H. microstoma*. Thus although *Vampirolepis microstoma *[12] represents the most recent formal taxonomic assignment of the species, few investigators have adopted this name, and most reports refer to it as either a member of the genus *Hymenolepis*, or with less frequency, *Rodentolepis*. In our view, a natural circumscription of hymenolepid species will not be attained without the application of molecular data [15].

To this end, Haukisalmi *et al*. [8] recently used 28S rDNA to analyze phylogenetic relationships among 32 hymenolepidid species from rodents, shrews and bats, showing that both *Hymenolepis *and *Rodentolepis *represented paraphyletic assemblages. Although their work assigned *H. microstoma *to a 'Rodentolepis' clade, the lack of resolution and widespread paraphyly of the taxa in their analyses indicate that greater taxonomic representation and more robust data are needed before such nomenclatural circumscriptions can be made reliably. We therefore follow Blanchard [5] in recognizing the mouse bile duct tapeworm as a member of the genus *Hymenolepis*, employing the most common name in usage, whilst appreciating that a more comprehensive understanding of hymenlepidid interrelationships is likely to warrant generic reassignment.

Here we provide a description of a 'Nottingham' strain of *H. microstoma *based on light and scanning electron microscopy of laboratory-reared specimens from the same culture used to characterize the genome. History of the isolate, dating back to the laboratory of C. P. Read [1], suggests that it represents a model that has been widely employed and disseminated within the parasitological community for over 50 years, making the genome data directly relevant to a significant pre-existing literature on its biology.

## Results

### Description of *Hymenolepis microstoma *(Nottingham strain)

*Hymenolepis microstoma *(Dujardin, 1845) Blanchard, 1891

#### Recorded synonyms

*Taenia microstoma *Dujardin, 1845; *Cercocystis tenebrionis *Villot, 1882; *Cysticercus tenebrionis *(Villot, 1882) Leuckart, 1886; *Cysticercus taenia-microstomae *Dolly, 1894; *Cysticercoides tenebrionis *(Villot, 1882) Braun, 1898; *Scolex *(= *Onchoscolex*) *decipiens *(Diesing, 1853) Joyeux and Kobozieff, 1928; *Rodentolepis microstoma *(Dujardin, 1845) Spasskii, 1954; *Vampirolepis microstoma *(Dujardin, 1845) Schmidt, 1986.

#### Common name

mouse bile duct tapeworm

#### Laboratory strain designation

'Nottingham'

#### Laboratory strain history

2005-present, The Natural History Museum, London (PDO); 1977-2005, University of Nottingham, UK (Prof. Jerzy Behnke); 1964-1977, University of Glasgow, UK (Prof. Adrian Hopkins); before 1964, Texas Rice University, USA (Prof. Clark P. Read).

#### Laboratory hosts

flour beetles (*Tribolium confusum*) and BKW outbred conventional mice (*Mus musculus*).

#### Voucher specimens

20 whole-mounted specimens (BMNH 2010.12.8.1-20), 22 slides of histological sections of adult worms (scolex and neck: BMNH 2010.12.8.21-30; immature strobila: BMNH 2010.12.8.31-36; mature strobila: BMNH 2010.12.8.37-42), and 12 whole and partial specimens prepared for SEM, retained by the corresponding author.

#### No. chromosomes

12 diploid, all acrocentric [16,17]

#### Genome size

~140 Mb (haploid)

#### Genome data

http://www.sanger.ac.uk/resources/downloads/helminths/hymenolepis-microstoma.html

#### Description

**Figure 1:**
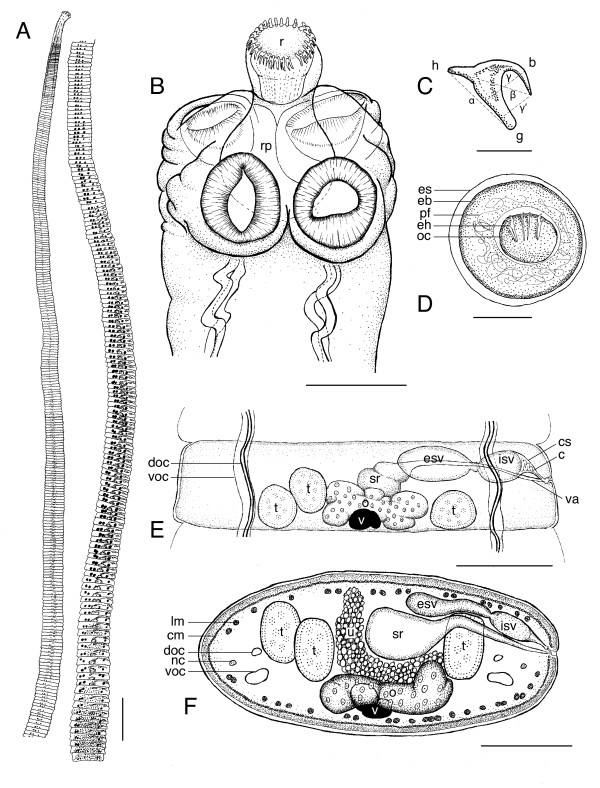
**Illustrations of adult *Hymenolepis microstoma *(Nottingham strain)**. A. Whole worm. B. Hook showing measurement vectors. C. Egg. D. Scolex. E. Mature proglottide. F. Cross section of mature proglottide. Abbreviations: b, blade; c, cirrus; cs, cirrus sac; doc, dorsal osmoregulatory canal; eb, embryophore; eh, embryonic hooks; es, eggshell; esv, external seminal vesicle; g, guard; h, handle; isv, internal seminal vesicle; nc, nerve cord; o, ovary; oc, oncosphere; pf; polar filaments; r, rostellum; rp, rostellar bulb; s, shell; sr, seminal receptacle; t, testis; u, uterus; va, vagina; voc, ventral osmoregulatory canal. Scale bars: A = 1 mm; B = 10 μm; C = 50 μm; D-F = 100 μm.

**Figure 2:**
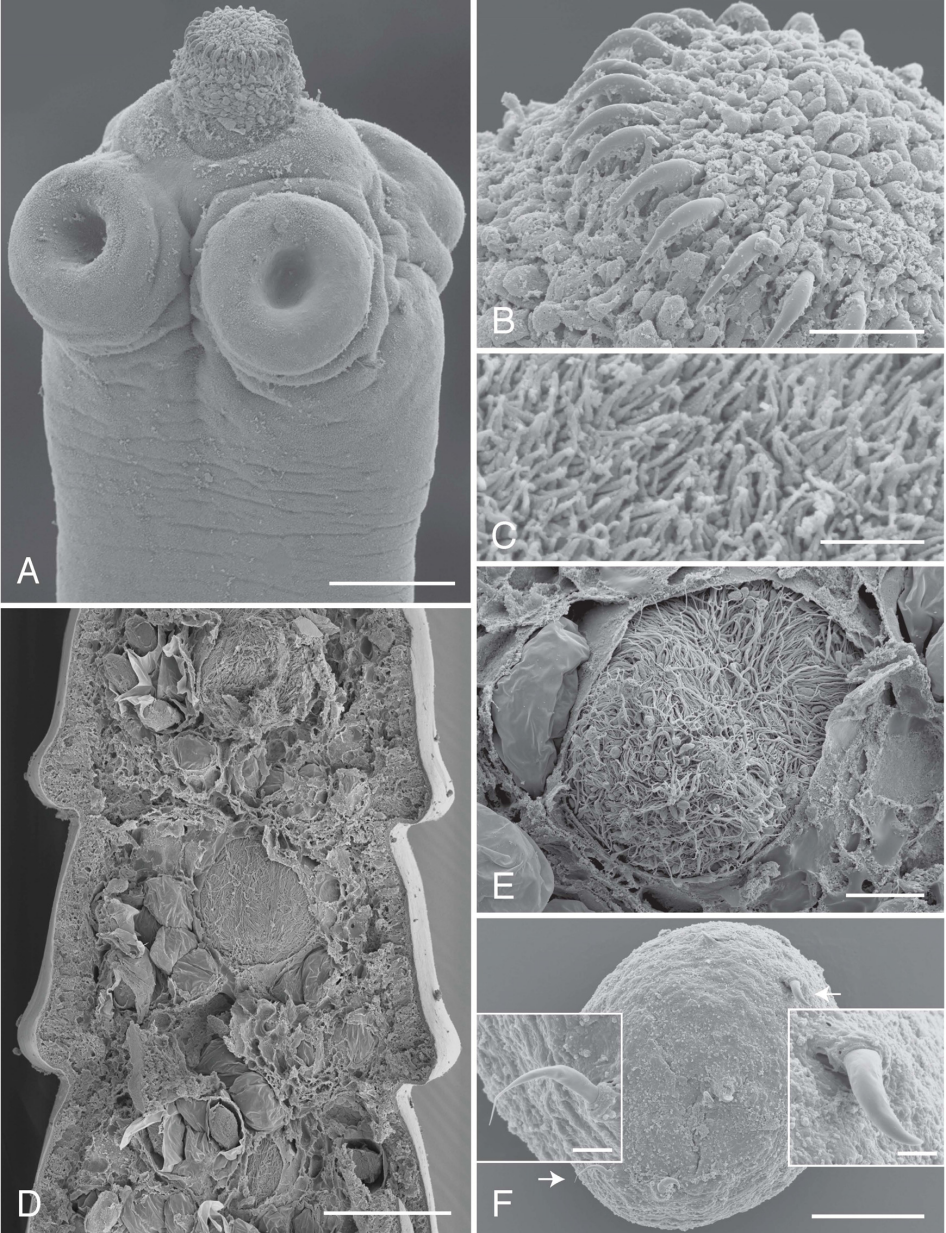
**Scanning electron micrographs of adult *Hymenolepis microstoma *(Nottingham strain)**. A. Scolex and rostellum. B. Rostellar hooks. C. Microtriches on the scolex. D. Internal view of gravid strobila. E. Seminal receptacle with spermatozoa surrounded by eggs. F. Three-day old transforming oncosphere showing larval hooks (arrows and insets). Scale bars: A = 50 μm; B = 5 μm; C = 2 μm; D = 100 μm; E-F = 20 μm (insets = 2 μm).

(based on 14-16 day old *in vivo *laboratory-reared specimens: 20 whole-mounted, 2 sectioned, and 12 specimens prepared for SEM; Figures. 1-2; all measurements are given as length × width in μm except where noted): worms anapolytic, weakly craspedote, 4.7 (2.5-8.1) cm long, with 659 (291-1,087) total segments (Figure 1A); scolex 138 (116-157) × 232 (204-284) with four muscular suckers 102 (79-129) × 96 (76-113) (Figure 1B). Rostellum 38 (26-52) × 71 (51-75) with an irregular surface lacking microtriches (Figures. 2A, B), armed with 25 (22-26) hooks, retractable into contractile rostellar pouch 104 (83-139) × 101 (79-140) (Figure 1B). Hooks cricetoid; α = 13.9, β = 12.3, γ = 6, γ' = 4.4 (Figure 1C). Width at level of neck 175 (94-225). Immature segments 62 (38-83) × 404 (437-463), mature segments 117 (70-167) × 729 (360-887), gravid terminal segments 164 (131-262) × 1,160 (454-1,426). Paired osmoregulatory vessels and longitudinal nerve cords lateral (Figure 1E, F). Entire worm covered by short (~2 um), uniform and densely packed filiform microtriches [18] (Figure 2C).

Male system consisting of three spherical to oval testes 72 (51-114) × 78 (61-115), arranged one poral and two aporal (occasionally reversed in individual proglottides). Vas deferens expands to form an external seminal vesicle 105 (73-195) × 56 (38-93) (Figure 1E). Cirrus pouch ovoid 58 (45-99) × 153 (97- 302), enclosing coiled cirrus and internal seminal vesicle, 52 (38-93) × 94 (72-195). Female system consisting of a central lobed ovary 63 (34-103) × 234 (130-360) partially overlapping a compact vitellarium 38 (41-94) × 56 (32-68). Seminal receptacle median. Vagina 299 (197-431) × 13 (9-18) situated ventral to male system (Figure 1F). Common genital pore dextral, unilateral, near mid-point of margin. Eggs thin-shelled (Figure 2E), enclosing embryophore with 3 polar filaments and oncosphere with embryonic hooks arranged in parallel (Figure 1D).

### Remarks

*Hymenolepis *species exhibit the well-documented 'crowding effect' in which overall size and egg production are inversely related to the intensity of infection [19]. Consequently, size is dependent not only on the age of the worms, but on the number of worms present in the host, and cannot be used diagnostically [20]. Crowding in *H. microstoma *has been shown to decrease linear growth, egg production and the rate of proglottide formation [21]. Moreover, we chose to document gravid adult specimens at an age and size most useful for laboratory manipulations in which larger worms pose unnecessary practical problems (e.g. assays involving whole mount *in situ *hybridization or in vitro culture). de Rycke [22] showed that *H. microstoma *is in rapid state of growth starting around 12-14 days post-infection in *Mus musculus*, and whereas our length measurements correspond to those reported by de Rycke for the relevant age class (see Table 1), they are obviously less comparable to reports based on older specimens, such as those stemming from natural infections.

**Table 1:** Growth of *Hymenolepis microstoma* in *Mus musculus* (summarized from de Rycke [22])

**Days p.i**.	Avg. length (mm)	Development and position in gut
**1-2**	0.25-0.50	no external segmentation or genital anlagen; worms localized in the first 10-20 cm of the intestine
**3**	1.58	some internal segmentation; appearance of genital anlagen; worms localized in the first 10 cm of the intestine
**4-5**	3.40-3.85	external segmentation and male & female genital anlagen discernable; worms localized in the bile duct
**6**	5.85	testes in few segments
**7**	9.15	testes mature
**8**	13.50	early-mature to mature proglottides
**9-10**	17-20.50	all proglottides mature
**11**	27	disappearance of female glands; few pre-oncospheres
**12**	36	pre-oncospheres, no hooks
**13**	46.5	semi-gravid proglottides
**14**	62.5	near gravid proglottides
**15-16**	94-129	gravid proglottides

## Discussion

### Life history

*Hymenolepis microstoma *is most probably cosmopolitan in distribution [20] and is not known from human infections outside of a single report in which mixed infections of *H. nana *and *H. microstoma *were identified in four individuals from a remote region of Western Australia [23]. Reported natural definitive hosts include a large range of rodent genera that include mice (e.g. *Apodemus *Kaup, *Dendromus *Smith, *Leggada *Gray, *Mastomys *Thomas, *Mus *L.), gerbils (*Meriones *Illiger) and voles (*Microtus *Schrank) [9,12,24]. Infections in rats is controversial: whereas Joyeux and Kobozieff [25] reported successful infection of laboratory rats, Dvorak *et al*. [20] found rats to be refractory to *H. microstoma*, and Litchford [24] showed that rats became refractory with age. Similarly, although infections can be established in golden hamsters (*Mesocricetus *Nehring), they result in underdeveloped worms and cause severe pathology to the host [20,24]. Dvorak *et al*. [20] demonstrated that mice could not be infected via eggs, as is the case with *H. nana *(ie. auto-infection) [26]. However, in congenitally athymic mice, Andreassen *et al*. [27] found that autoinfection was possible, showing that oncospheres penetrated the intestinal tissues and developed into cysticeroids that subsequently excysted and developed normally in the bile duct and duodenum, in a manner similar to the direct cycle of *H. nana*. Autoinfection of BALB/c mice was also implied by the detection of stage-specific antigens [28].

**Figure 3:**
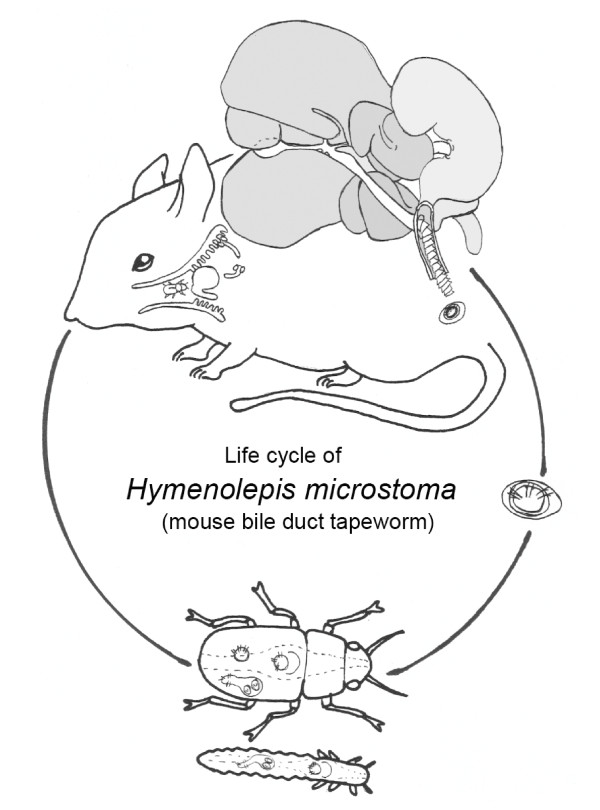
**Life cycle of *Hymenolepis microstoma***. Infected adult or larval beetles (e.g. *Tribolium confusum*) are consumed by rodents (e.g. *Mus musculus*), releasing the cysticercoids which excyst and locate in the bile duct before commencing strobilation. Gravid adult worms develop in 12-14 days in vivo and release embryonated eggs in the duodenum that are expelled with the host faeces. Oncosphereal larvae are released when the eggs are consumed by beetles, allowing them penetrate the gut wall and metamorphose into patent cysticercoids in the haemocoel (apx. one week). Illustration adapted from Olsen [63].

The life history of *H*. *microstoma *(Figure 3) has been described in detail previously [20,25,29] and is typical of other hymenolepid species, save its unusual location in the bile duct of the mammalian host. In brief, eggs containing patent oncospheres are expelled with faeces into the environment and may be ingested by either the adult or larval stage of an appropriate beetle host (e.g. *Tribolium confusum*, *T. castaneum*, *Tenebrio molitor*, and *Oryzaephilus surinamensis*). Oncospheral larvae (~20 μm; Figure 1D; Figure 2F) are released from their thin shells (Figure 2E; n.b. appearing as a 'hymen' via light microscopy and the eponym of the genus) through the action of the host mouthparts, and after ingestion use their three pairs of hooks and proteolytic secretions [30] to enter the haemocoel. There they undergo a complete metamorphosis, reconstituting their bodies into cycsticeroid larvae [31] in approximately seven days, the phases of which have been documented by both Voge [32] and Goodchild and Stullken [33]. Upon infection of the definitive host, the combination of pepsin and HCl in the stomach act to dissolve the larval membranes, and juvenile worms are then activated in the duodenum in response to trypsin and bile salts. de Rycke [22] described adult growth and organogenesis in *Mus musculus *(summarized in Table 1): in the first three days the juveniles move anteriorly in the upper 20% of the small intestine and duodenum before establishing permanently in the bile duct, where they commence strobilation. Within approximately 14 days terminal segments are gravid and most of their strobila extends outside of the bile duct and into the duodenum. Thus the entire life cycle, from egg to gravid adult, can be completed in the laboratory in only three weeks. Although the germinative ('neck') region of tapeworms has the potential for 'immortality' as demonstrated in *H. diminuta *by Read [34], infections of *H. microstoma *in mice persist for an average of six months, whereas those in the intermediate host can remain infective for the life of the beetle (> one year).

### The *Hymenolepis *genome

Through collaboration with The Wellcome Trust Sanger Institute, a draft genome of *H*. *microstoma *derived from the cultures described herein is now publically available: http://www.sanger.ac.uk/resources/downloads/helminths/hymenolepis-microstoma.html. The latest assembly (October 2010) includes more than 40× coverage of the estimated 140 Mb haploid genome and is based on data produced by a combination of Roche 454 and Illumina Solexa next-generation sequencing technologies. Gene annotation is presently being conducted using a combination of RNA-Seq [35] and automated gene prediction tools, revealing intron-exon structures and other aspects of their genomic organization, and additional tools are being used to characterize non-coding regions (M. Zarowiecki and M. Berriman, *pers. comm*.).

*Hymenolepis microstoma *is one of four tapeworm species to have complete genomes characterized: a reference genome of *Echinococcus multilocularis *Leukart, 1863 and draft genome of *E. granulosus *(Batsch, 1786) have been produced by the Sanger Institute (available from http://www.sanger.ac.uk/resources/downloads/helminths/) in collaboration with Profs. Klaus Brehm and Cecelia Fernandez, respectively, and a consortium in Mexico are currently working to characterize the genome of *Taenia solium *L., 1758 [36]. These data herald the beginning of the genomic era in cestodology and are already accelerating advances in our understanding of tapeworm biology and infection. At present the only published platyhelminth genome is that of the human blood fluke, *Schistosoma mansoni *Sambon, 1907 [37]. However, genome data for *Schistosoma *Weinland, 1858 and *Echinococcus *Rudolphi, 1801, as well as the free-living flatworm models *Schmidtea mediterranea *Benazzi, Baguna, Ballester, Puccinelli and Del Papa, 1975 [38] and *Macrostomum lignano *Ladurner, Scharer, Salvenmoser and Rieger, 2005 (http://www.macgenome.org/), have been available for some time and full reports on the characteristics of all of these genomes, including that of *H. microstoma*, are expected soon.

### Model systems in the genomic era of cestodology

Of the three *Hymenolepis *species that have been employed in laboratory research, most literature concerns the rat tapeworm *H. diminuta*, followed by the medically important dwarf tapeworm, *H. nana*, and finally by the mouse bile duct tapeworm, *H. microstoma*. As a model for research in the genomic age, however, *H. microstoma *has advantages over both of these alternative systems. For example, compared to *H. diminuta*, it is both smaller and mouse-hosted, enabling smaller, and thus less expensive, assay sizes (e.g. for RNAi), as well as less expensive animal costs, whereas the mouse-hosted *H. nana *is both a human pathogen (albeit controversy persists regarding the conspecficity of human and mouse strains) and capable of infecting other laboratory animals through faecal contamination via its direct life cycle [26]. Moreover, whereas *H. nana *survives only weeks in the mouse host [39], *H. microstoma *persist for ~6 months and thus require less frequent passage. Although the smaller size of *H. nana *would be preferable for assays, on balance *H. microstoma *provides the best practical solution for contemporary research programmes that wish to employ a tapeworm model providing easy access to all stages of their life cycle at minimal expense and risk to human and animal health.

Completion of the *H. microstoma *life cycle *in vitro *from egg to gravid adult was demonstrated in the 1960s and 70s by De Rycke and Berntzen [40], Evans [41,42] and Seidel [43,44], but to our knowledge no report of research employing these techniques has been published subsequently. Our initial attempts to follow these protocols for the cultivation of adult worms resulted in only limited growth (3× increase in length) without the onset segmentation (unpub. data). However, as many of the reported media used by previous authors are no longer commercially available, more work is needed to develop contemporary protocols for *in vitro *culture. Among the most advanced *in vitro *systems available for tapeworm research today has been developed by Brehm and colleagues for *Echinococcus *[45-48], the genus on which most of our understanding of tapeworm molecular biology is based [49]. Development of an axenic culture system of the hydatid stage of *E. multilocularis *has allowed them to introduce transgenic and functional genomic techniques (e.g. RNAi) to cestodology, and their system is currently being used to pioneer research on stem-cells and developmental biology in parasitic flatworms [45,50]. Although not yet supported by genome characterization, another currently employed *in vitro *system is that of *Mesocestoides *Vaillant, 1863 [e.g. [51]] which are readily maintained in the larval tetrathyridial stage [31] and can increase their numbers in culture via asexual fission [52]. Adult worms have also been grown *in vitro *and induced to strobilate through the addition of bile salts [53]. However, as with species of *Echinoccocus *and *Taenia*, *in vivo *development of strobilar stages of *Mesocestoides *is prohibited by the legalities and expense of maintaining large vertebrate hosts in the laboratory. Rodent hosted *Hymenolepis *species therefore remain the most convenient systems for research on the biology of adult tapeworms, and for this reason we have been developing *H. microstoma *as a model to study the development and evolution of tapeworm segmentation [54].

Although the basic framework of cestode evolution has been revealed by previous molecular studies [55-58] and the interrelationships of select groups are now well resolved [59-61], there has yet to be a comprehensive molecular phylogenetic study of the largest and most important group of tapeworms with regard to human and animal health, the Cyclophyllidea. All of the tapeworm species for which genomes have been characterized thus far belong to this order and thus it is especially important that we elucidate the relative phylogenetic positions of the 350+ described genera [14]. Such knowledge will provide an evolutionary underpinning for comparative genomic studies within the group and allow us to identify the sister lineages whose genomes share the closest evolutionary histories to the species for which full genome data are now available.

## Methods

A seed culture of *Hymenolepis microstoma *infected beetles was obtained from Nottingham University in 2005 courtesy of Prof. Jerzy Behnke and subsequently maintained *in vivo *at the Natural History Museum (London) using flour beetles (*Tribolium confusum*) and BKW outbred conventional mice (full protocols can found at http://www.olsonlab.com; please contact the corresponding author to enquire about seed cultures). Gravid, 14-16 day old specimens were removed from the bile ducts and duodenum of mice and quickly swirled in near-boiling 0.85% saline for ~4 secs to fully extend the worms prior to fixation in cold 4% paraformaldehyde overnight at -4 C. Whole-mounted specimens were dehydrated in a graded ethanol series, stained using Gill's haematoxylon or left unstained, cleared in beachwood creosote and mounted in Canada balsam. Sections were prepared by paraffin embedding using standard histological techniques and stained with Mayer's Haemalum [62]. Measurements and illustrations were made under differential interference contrast on a Leica DM5000B compound microscope equipped with a camera lucida and digital documentation system. Specimens used for SEM were dehydrated as above, critically-point dried, sputter-coated with gold/palladium and viewed on a JEOL XL30 scanning electron microscope. Internal structures were imaged by SEM by cutting worms crudely using a razor blade.

## Competing interests

The authors declare that they have no competing interests.

## Authors' contributions

PDO designed the study and drafted the manuscript. LC carried out research. Both authors read and approved the final manuscript.
